# Deregulation of Lipid Metabolism: The Critical Factors in Ovarian Cancer

**DOI:** 10.3389/fonc.2020.593017

**Published:** 2020-10-19

**Authors:** Zhaodong Ji, Yan Shen, Xu Feng, Yue Kong, Yang Shao, Jiao Meng, Xiaofei Zhang, Gong Yang

**Affiliations:** ^1^ Cancer Institute, Fudan University Shanghai Cancer Center, Shanghai, China; ^2^ Department of Oncology, Shanghai Medical College, Fudan University, Shanghai, China; ^3^ Department of Pharmacy, Nantong Health College of Jiangsu Province, Nantong, China; ^4^ Department of Gynecology, Shanghai First Maternity and Infant Hospital, Tongji University School of Medicine, Shanghai, China; ^5^ Central Laboratory, The Fifth People’s Hospital of Shanghai Fudan University, Shanghai, China

**Keywords:** ovarian cancer, lipid metabolism, potential target, fatty acid synthesis, metabolic enzyme

## Abstract

Ovarian cancer is one of the most malignant gynecological cancers around the world. In spite of multiple treatment options, the five-year survival rate is still very low. Several metabolism alterations are described as a hallmark in cancers, but alterations of lipid metabolism in ovarian cancer have been paid less attention. To explore new markers/targets for accurate diagnosis, prognosis, and therapeutic treatments based on metabolic enzyme inhibitors, here, we reviewed available literature and summarized several key metabolic enzymes in lipid metabolism of ovarian cancer. In this review, the rate limiting enzymes associated with fatty acid synthesis (FASN, ACC, ACLY, SCD), the lipid degradation related enzymes (MAGL, CPT, 5-LO, COX2), and the receptors related to lipid uptake (FABP4, CD36, LDLR), which promote the development of ovarian cancer, were analyzed and evaluated. We also focused on the review of application of current metabolic enzyme inhibitors for the treatment of ovarian cancer through which the potential therapeutic agents may be developed for ovarian cancer therapy.

## Introduction

Ovarian cancer, as one malignant gynecological cancer, is the eighth leading cause in cancer-related death around world (1). According to the latest statistical cohort from the Surveillance, Epidemiology and End Results (SEER) in 2017, there was an annual incidence of 11.6 cases/100,000 women per year, with an estimated 224,940 women living with this disease in the world (2). Because of hidden symptoms and lack of effective diagnostic methods, about 70% of patients are diagnosed in advanced stage when they receive treatment for the first time (3), which underlines the status of ovarian cancer as a serious public health concern for women. Based on the various research and epidemiological investigations, the pathogenesis of ovarian cancer mainly include viral infection, endocrine disorders, genetics, and environmental pollution (4–7). Ovarian cancer is characterized by widespread and rapid metastasis in the peritoneal cavity, which facilitates metastatic dissemination and poor disease progression. Malignant ascites constitute a unique tumor microenvironment providing a physical structure for the accumulation of many components. A large number of cancer-promoting components such as cytokines, proteins, and metabolites in ascites are reported to promote cancer invasion and resistance to chemotherapy through surface-specific receptors on tumor cells (8–10). Meanwhile, the malignant progress of ovarian cancer also brings a series of changes in its own metabolism including glycometabolism, lipid metabolism, and amino acid metabolism, which may further strengthen the malignancy of the disease (11–14).

Lipids, as important nutrients for the body, are a class of water-insoluble substances including triacylglycerol, glycerol phosphates, sterols, and sphingolipids. In addition to providing a large amount of energy, lipids are also widely distributed in cellular organelles and used as biologically vital active molecules in a variety of signaling pathways to participate in process of inflammation, immunity, cell proliferation, and differentiation (15, 16). Four major routes demonstrate how lipids are routed and used in the cell: uptake, lipogenesis, storage, and degradation. The lipogenesis refers to the fatty acid synthesis pathway and the mevalonate pathway, the latter mainly leading to cholesterol and isoprenoid synthesis. The important raw material for the *de novo* synthesis of fatty acids is acetyl-CoA, which comes from two approaches: one is citric acid from the tricarboxylic acid cycle. Citrate is transported across the inner mitochondrial membrane by the transport protein CIC (citrate carrier) and then catalyzed by ATP-citrate lyase (ACLY) to produce acetyl-CoA and oxaloacetate. The other is that cells uptake acetic acid directly from the outside and catalyze the production of acetyl CoA through acetyl CoA synthetase (17, 18). Deregulation of lipid metabolism including the increasing *de novo* synthesis and degradation of fatty acid often occurs in a variety of cancer diseases, which could provide cancer cell a strong support for proliferation, invasion and metastasis. A large number of studies have found that in multiple cancers, the expression and activity of various enzymes involved in the synthesis and catabolic pathways of fatty acids (phospholipids and cholesterol) are significantly up-regulated. In addition, other lipid-metabolizing enzymes such as lipoxygenase (LOX) and cyclooxygenase (COX) gradually become cancer research hotspots in recent years. Oncogenes highly expressed in cancer cells can activate the PI3K/AKT/mTOR signaling pathway to allow the related proteins such as ErbB2 and HIF-1 to promote the expression of lipid synthetases (19–21).

Lipid droplets (LDs) occurring in specialized cytoplasm are considered to be special lipid storage organelles because they can synthesize and store triglycerides. LDs are composed of a core of neutral lipids, surrounded by phospholipids and cholesterol, and specific proteins are embedded or associated with their surroundings. More and more evidence shows that LDs are not only passive reservoirs of lipids, but are actually dynamic organelles that play a central role in lipid and energy metabolism (22).

Since fatty acids are essential for cancer malignant progression, the availability of rate limiting enzymes in lipid metabolism could be therapeutic targets. Lipid metabolism could be regulated by suppressing fatty acid synthesis, accelerating fatty acid degradation *via* oxidation, diverting fatty acid to storage, retarding fatty acid release from storage, and blocking fatty acids intake (23). Limiting lipid metabolism through these mechanisms could be accomplished in alone or in a combinatorial manner, which could pave the way for the therapy of ovarian cancer (**Table 1**). This article summarizes the effects of lipid metabolism disorders in ovarian cancer from two aspects: exogenous lipid metabolism and endogenous lipid metabolism.

**Table 1:** Chemical Inhibitors targeting enzymes of lipid metabolism in ovarian cancer.

Enzyme	Chemical inhibitor	Notes	Pathway	Models(animal/cell line)	Reference
FASN	Orlistat	Reduce proliferation and promotes apoptosis Platinum resensitization	—–	Mouse A2780	(24)
	Compound 34	Inhibits proliferation	—–	A2780	(25)
	Cerulenin or C75 (A cerulenin derived)	Induce apoptosis Platinum resensitization	Receptor/PI3K/mTORC1	SKOV3, OVCAR3, A2780, HOC-7	(26) (27)
	TVB-3664	Reduce tubulin palmitoylation Inhibit proliferation	PI3K/AKT/mTOR β-catenin signal	OVCAR5/8	(28)
	C93	Induce apoptosis	NAC1-FASN	SKOV3, A2780, OVCAR3	(29)
	TVB-3166	Induce apoptosis and anchorage-independent cell growth	PI3K/AKT/mTOR	OVCAR5/8	(28)
SCD1	A939572	Cause cell death	——	SKOV3 Mouse	(30)
	CAY10566	Reduce the lipid unsaturation levels in OC spheroids	STAT/NFκB/SCD1	OVCAR5, COV362	(31)
	MF-438	Induce apoptosis and ferroptosis	—	SKOV3	(30)
	CAY10566	Induce apoptosis and ferroptosis	—–	SKOV3	(30)
ACC1	TOFA	Suppress the proliferation and induce apoptosis. Inhibit growth	Down-regulated the expression of cyclin D1, CDK4 and Bcl-2 Caspase-3 was cleaved and activated.	COC1 Mouse	(32)
MAGL	JZL184	Decrease cancer cell migration	—–	SKOV3, OVCAR3	(33)
CPT	Etomoxir	Reduce tumor growth rate, ascites production	—–	Mouse	(34)
5-LO	Zileuton	Reduce the MMP-7 expression and the number of macrophages infiltrating	P38 pathway	Mouse	(35)
COX2	Celecoxib	Reduce invasion Inhibit proliferation Induce cell cycle arrest in G0/G1 and apoptosis Inhibit tumor growth	COX2/Snail/E-cadherin	1. SKOV3, ES-2 Hey, IGROV1 2. Mouse	(36) (37)
	Berberine	Inhibit the chemotherapy‐induced repopulation of ovarian cancer cells	Caspase3/iPLA2/AA /COX2/PGE2	SKOV3	(38)
FABP4	BMS309403	Reduce tumor burden Increase the sensitivity of carboplatin	—–	Mouse HeyA8, SKOV3	(39)

## Endogenous Lipid Metabolism

In lipid metabolism of ovarian cancer cells, many metabolic enzymes are abnormally expressed, which can cause lipid metabolism disorders by participating in processes that affect lipid synthesis or degradation, thereby to provide raw materials and energy for cancer development. At present, the combination of inhibitors of rate limiting metabolic enzymes with first-line chemotherapy agents has become a new strategy for treatment of ovarian cancer.

### Rate Limiting Enzymes in Fatty Acid Synthesis

#### ATP-Citrate Lyase (ACLY)

**Figure 1:**
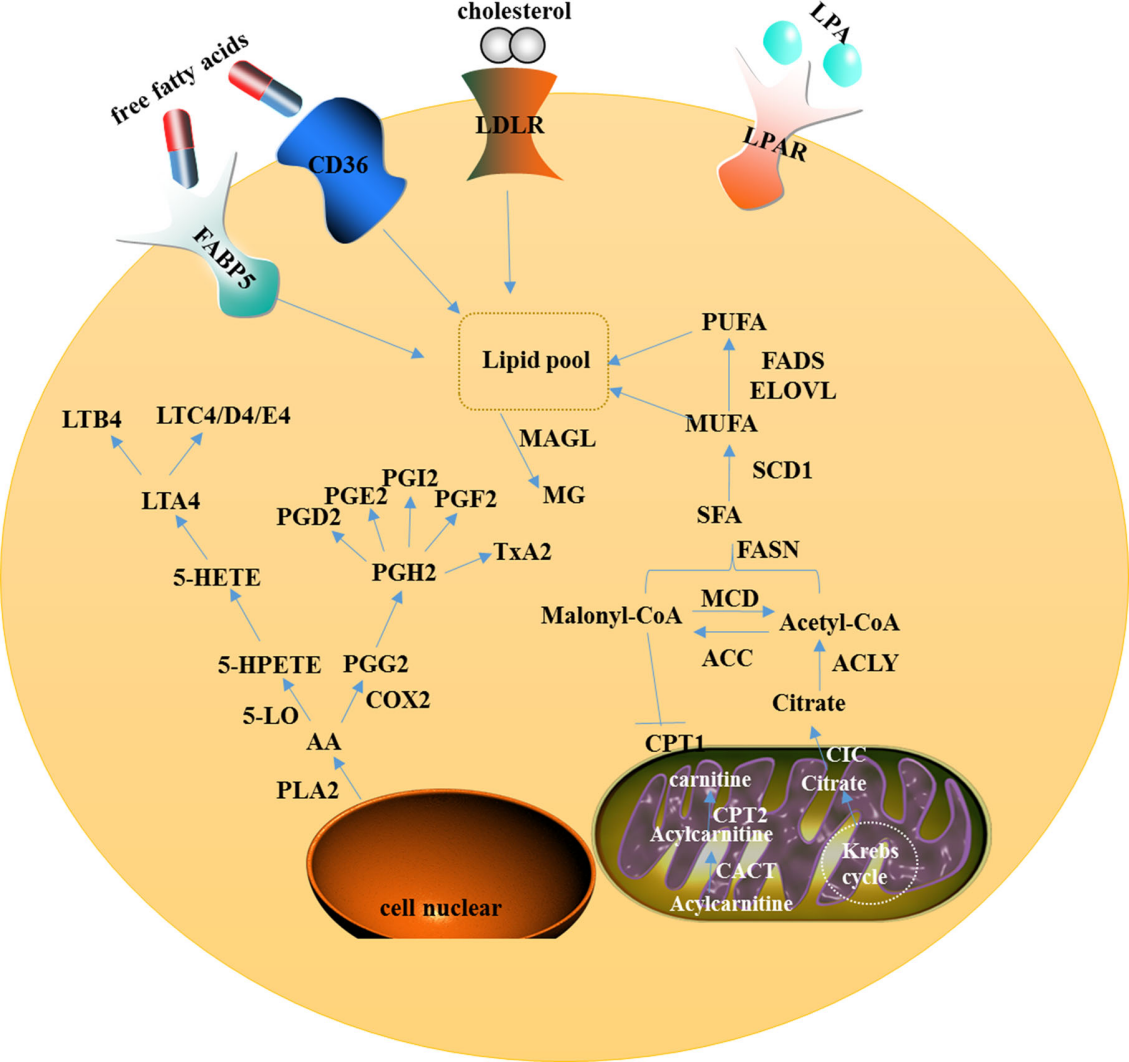
A model showing intracellular lipid metabolism and AA metabolism in ovarian cancer. In the cytoplasm of the cell, fatty acid metabolism includes uptake, *de novo* lipogenesis, and degradation. AA can be metabolized *via* two major pathways, namely the lipoxygenase pathway and the cyclooxygenase pathway. In mitochondria, CIC promotes the efflux of citrate from the mitochondria to the cytosol and CACT catalyzes acylcarnitine to translocate through the inner mitochondrial membrane.

ATP-citrate lyase (ACLY), the upstream enzyme in fatty acid biosynthesis, functions physiologically to catalyze the six-carbon citric acid from the tricarboxylic acid cycle, either from glucose by glycolysis or glutamine, to oxaloacetate and acetyl on the cytosolic side, which provides raw materials for the synthesis of fat acid and cholesterol (**Figure 1**). Therefore, it is considered as a bridge connecting glycometabolism and lipid metabolism (40). The AKT-mediated phosphorylation of ACLY could promote histone acetylation in cancer cells and immune cells to response to the oncogenic and cytokine-induced signaling, while ACLY is transcriptionally regulated by SREBP1 (sterol regulatory element binding transcription protein-1) (41, 42). In addition, other substances such as insulin, glucagon, and TGF-β can promote the phosphorylation of ACLY.

Wang et al. found that ACLY expression was higher in malignant tissues than that in normal ovarian tissues. Immunohistochemical analysis showed that the increased expression level of phosphorylated ACLY in ovarian cancer tissues was related to cancer grade, FIGO stage, and poor prognosis. Mechanismlly, by knockdown of ACLY expression could inhibit the proliferation of ovarian cancer A2780 cells and cause G1 phase arrest (43, 44), suggesting that ACLY promoted cancer cell proliferation through the regulation of cell cycle.

The ubiquitin-proteasome controls protein degradation and regulatory functions. Ubiquitin-specific proteases (USPs) are the largest family of deubiquitin, which can catalyze the removal of ubiquitin from different target proteins to regulate cell function. Studies have reported that ubiquitin-specific peptidase 13 (USP13) was the main regulator of ovarian cancer metabolism (45). ACLY can be one of deubiquitinase target proteins of USP13, removing K48-related ubiquitination on ACLY to improve the stability of ACLY. The *in vitro* experiments found that the inhibition of USP13 expression could significantly inhibit the progression of ovarian cancers and enhance the sensitivity of cancer cells to treatment with PI3K/AKT inhibitors. Therefore, the researchers proposed that ACLY may play an important role in the USP13-mediated deubiquitination to promote cancer development (45, 46).

### Acetyl-CoA Carboxylase (ACC)

The first committed step of fatty acid synthesis is mediated by acetyl- ACC, which in mammals is encoded by two subtype enzymes ACC1 (Acetyl-CoA Carboxylase Alpha) and ACC2(Acetyl-CoA Carboxylase Beta) (47). ACC1 is generally expressed in lipogenic tissues, and the ACC1-generated malonyl-CoA is utilized for the synthesis of fatty acids in cytosol. In contrast, ACC2 is highly expressed in heart and muscle and to a lesser expressed in liver. Unlike ACC1 promoting fatty acid synthesis, ACC2 is anchored at outer membrane of mitochondria in subcellular where localized malonyl-CoA production blocks carnitine palmitoyltransferase-1 (CPT1) function to prevent fatty acids from entering the mitochondria to undergo fatty acid oxidation (48). ACC is a biotin-dependent multi-domain enzyme, which has biotin carboxylase (BC) and carboxyl transferase (CT) activities in most eukaryotes. In regard to these two enzyme activities, BC catalyzes the ATP-dependent carboxylation of biotin with bicarbonate as a CO2 donor, and CT promotes the transfer of carboxyl groups from biotin to acetyl CoA. In recent years, ACC activity is tightly regulated by reverse phosphorylation and gene expression. The phosphorylation of ACC by adenosine monophosphate-activated protein kinase (AMPK) has been identified (49). Notably, ACC1 has been shown to be elevated in a number of cancers, including liver cancer, lung cancer, breast cancer, and pancreatic cancer. Inhibitors targeting ACC1 were shown to reduce cell proliferation through inhibiting fatty acid synthesis (50, 51).

TOFA, an allosteric inhibitor of ACC1, was reported to suppress the proliferation of ovarian cancer *via* arresting the cells in G0/G1 cell cycle phase and inducing apoptosis (32). Meanwhile, TOFA could inhibit growth of ovarian cancer xenograft in mice. One study based on a randomized multicentre phase 3 trial (MITO2) found that carboplatin/PLD might be more effective than carboplatin/paclitaxel to ovarian cancer patients in the presence of pACC overexpression, suggesting that ACC might be a new biomarker for personalizing the choice of chemotherapy regimen in ovarian cancer (52).

### Fatty Acid Synthase (FASN)

FASN is a key enzyme for endogenous fatty acid synthesis. This cytosolic enzyme catalyzes the synthesis of 16-carbon palmitic acid by malonyl-CoA and acetyl-CoA under the action of reducing coenzyme II (53) (**Figure 1**). In normal condition, the physiological function of FASN is to convert excess carbohydrates into fatty acid, which will be further esterified into triacylglycerols, and finally stored or supplied for energy through β oxidation. As a downstream effector, FASN could be activated by the PI3K/AKT/mTOR signaling pathway and the transcription factors such as SREBP-1, ZBTB7A, and p53 (54, 55). FASN is highly expressed in ovarian cancer tissues and is associated with poor prognosis and survival rate (56). Because the majority of cancers rely on the FASN-mediated *de novo* fatty acid synthesis pathway, FASN could be an attractive therapeutic target, and inhibition of FASN has shown antitumor effects in ovarian cancer (25, 57).

In tumor cell lines, FASN overexpression was found to cause chemotherapy resistance induced by culture in drug-containing media. This means that FASN may be involved in chemoresistance of cancer cells. O Bauerschlag et al. treated HEY cells with cerulenin, an inhibitor of FASN, and found that cerulenin markedly decreased FASN expression and cell viability, and induced apoptosis. Unlike combined administrations, sequential cerulenin, and cisplatin treatment profoundly reduced cisplatin’s half maximal inhibitory concentration in a cisplatin-resistant cell line, suggesting that cerulenin had reinduce platinum sensitivity (26). Papaevangelou et al. conducted a metabolite analysis and histopathology of ovarian cancer xenograft mice treated with the combination of the anti-obesity drugs orlistat and cisplatin, and found that orlistat reduced cancers by inhibiting FASN. At the same time, cisplatin reduced the β-oxidation of fatty acids, and combined therapy delayed the cisplatin-resistant ovarian cancer cell growth and induced apoptosis. The combination therapy of the two drugs also reduced glycometabolism, biosynthesis of nucleotides and glutathione, and β-oxidation of fatty acids (24).

Overexpression of FASN was also reported to be associated with tumor cell proliferation, metastasis, poor prognosis, and high risk of recurrence in breast cancer, prostate cancer and gastric cancer (58–60). The FASN inhibitor TVB-3166 can destroy the lipid structure on membrane of cancer cell, inhibit lipid biosynthesis, and promote cancer cell apoptosis through the PI3K-AKT-mTOR and β-catenin signaling pathways in ovarian cancer. At the same time, this inhibitor can also block the expression of the oncogene c-Myc (28). Some studies have pointed out that FASN inhibitors could also induce the cell cycle arrest at S/G2/M and apoptosis of cancer cells, but only caused cell cycle deceleration without apoptosis for normal cells (61). Therefore, FASN is proposed as a metabolic marker for ovarian cancer proliferation.

Recently, some scholars have found that the abnormal activation of FASN can blunt the anti-tumor immunity of host (62). The clinical data showed that, in the advanced stage of ovarian cancer, the abnormally increased expression of FASN was positively correlated with the state of immunosuppression. The immunosuppression was manifested in the lower number and dysfunction of infiltrating T cells. Mechanistic studies have found that FASN activation in ovarian cancer cells can induce the resulting lipid accumulation at high concentrations in the tumor microenvironment. High expression of FASN in ovarian cancer cells also caused defects in the ability of dendritic cells to present antigens and prime T cells in ascites. To further explore FASN inhibition effect in anti-tumor immune response *in vivo*, the use of FASN inhibitors could partially restore the immune-stimulating activity of Tumor-Infiltrating DCs (TIDCs) and evoke protective anti-tumor immune responses.

### Stearoyl COA Desaturase (SCD)

Stearoyl COA desaturase (SCD) is an endoplasmic reticulum enzyme that promotes a balance of saturated fatty acids (SFA) and mono-unsaturated fatty acids (MUFA) in cell lipids. Specifically speaking, SCD catalyzes the synthesis of MUFA SFA, principally stearic acid (18:0) and palmitic acid (16:0), to their D9-monounsaturated counterparts, oleic acid (18:1) and palmitoleic acid (16:1; ref. 8) (63). These MUFAs are major components of cell membrane phospholipids and cholesterol esters. Two SCD isoforms SCD1 and SCD5 have been identified in human, whereas other four desaturases (SCD1-SCD4) share the same enzymatic function exist in mouse (64). Among of five isoforms, SCD1 is expressed ubiquitously among tissue with a 33-amino acid sequence at the N terminus that leads to the rapid degradation of this enzyme *via* an ubiquitin-dependent proteasome (65). It has been identified that the promoter of SCD1 contains several binding sites with the peroxisome proliferator-activated receptor (PPAR), NF-1, AP-2, and SREBP. The enzyme activity of SCD1 is either promoted by insulin, glucose, and fructose or inhibited by unsaturated fatty acids, ethanol, TNFα, IL-11, thyroid hormones, and some steroid hormones (66). Previous studies have shown that SCD1 was overexpressed in many malignant cancers to regulate cell proliferation, cell cycle, apoptosis, metastasis, and to modulate lipid metabolism through reducing fatty acid oxidation to foster lipogenesis (67).

Roongta et al. found that the expression of SCD1 was up-regulated in ovarian cancer tissues and stem cells (68). Inhibition of SCD1 expression can induce cancer cell death. Conversely, overexpression of SCD1 or exogenous addition of palmitoleic acid can protect cells from death. Ferroptosis is an iron-dependent oxidative damage causing cell death that greatly inhibits the growth of ovarian cancer cells (69). Overexpression of SCD1 protects cells from ferroptosis through the increase of monounsaturated fatty acids, whereas inhibition of SCD1 significantly enhances the anticancer effect of ferroptosis-inducers on ovarian cancer cell lines and xenograft mouse tumors (30).

Scattering microscopy was used to observe an increase in unsaturated fatty acid level in ovarian cancer stem cells, and a significant increase in the mRNA level of SCD1 was detected by qRT-PCR. However, when SCD1 inhibitors were used to treat the primary ovarian cancer stem cells, the stemness markers were down-regulated. In addition, the treatment of ovarian cancer stem cells with SCD1 inhibitors retarded the tumor growth of cells when injected into athymic mice. Further study demonstrated that NF-κB may directly regulate the transcription of SCD1 (31).

### Limiting Enzymes in Fatty Acid Degradation

Cancer cells usually stimulate the degradation of fatty acids to provide energy for proliferation, and this degradation process can be achieved through mitochondrial β-oxidation. Within mitochondria, fatty acids continuously undergo cyclical series of reactions to produce acetyl-CoAs that were fed into the Krebs cycle and supply energy to tissues in demand when glycogen store is out of service (70).

### Monoacylglycerol Lipase (MAGL)

Monoacylglycerol lipase, a member of the serine hydrolase superfamily, mainly functions as a key enzyme to catalyze the decomposition of monoacylglycerol into free fatty acids and glycerol (**Figure 1**). Furthermore, MAGL controls several physiological processes including pain and nociperception through hydrolysis of the endocannabinoid 2-arachidonoylglycerol (2-AG). MAGL was highly expressed in ovarian and breast cancer tissues, and identified to contribute to tumorigenesis and metastasis through up-regulation of free fatty acids (33). MAGL also promotes epithelial-mesenchymal transition (EMT) and may serve as a gene expression signature for cancer stem cells (71, 72).

Other studies also found that the multiple inhibitors of MAGL could inhibit the proliferation of ovarian cancer cells (73, 74). The knockdown of MAGL expression inhibited the proliferation, migration and invasion of ovarian cancer cells (33).

### Carnitine Palmitoyltransferase (CPT)

When cancer cells lack glucose, energy is generated through the increased β-oxidation. CPT is a key enzyme that catalyzes the conversion of long-chain fatty acids into acylcarnitine, which can be inhibited by malonyl-CoA (**Figure 1**). Two subtypes of CPT (CPT1 and CPT2) differently catalyze the decomposition of long-chain fatty acids and β-oxidation. CPT1 resides at the outer membrane of mitochondria and transports long-chain fatty acids into mitochondria for β-oxidation. CPT2 is located on the mitochondrial inner membrane and catalyzes the production of acyl-CoA from acyl-carnitine-derived acyl groups and free coenzymes to shuttle across the inner mitochondrial membrane CACT (carnitine acylcarnitine translocase), which helps acylcarnitine to translocate through the inner mitochondrial membrane and to be converted back to acyl-CoA for β-oxidation and energy substrate generation. Increasing studies have reported that β-oxidation abnormality can be induced through the high expression of CPT1 to promote cancer progression (75).

Three different CPT1 isozymes are identified. CPT1A is widely distributed in multiple tissues with stronger enzyme activity. CPT1B is mainly expressed in skeletal muscle cells and cardiac muscle cells, while CPT1C is mainly found in testis and central nervous tissues. With the improvements of metabolic studies, it has revealed that CPT1 may promote cancer cell proliferation and survival (76).

Shao et al. found that CPT1A was highly expressed in ovarian cancer cell lines and primary ovarian serous carcinomas. Analysis of database revealed that overexpression of CPT1A was associated with poor survival in ovarian cancer patients. Knockdown of CPT1A expression reduced the cellular level of ATP and induced the cell cycle arrest at G0/G1 in ovarian cancer cells, indicating that the CPT1A-mediated β-oxidation controlled the proliferation through regulating cell cycle process. Knockdown of CPT1A stimulated the phosphorylation of the transcription factor FOXO through the AMPK/p38/JNK signaling pathway and up-regulated P21 to arrest cell cycle (77).

Roy et al. found that overexpression of CPT1A can increase the β-oxidation of fatty acids and ATP levels to promote cancer cell proliferation. In contrast, Etomoxir, a specific inhibitor of CPT1A, can inhibit the proliferation of ovarian cancer cells (34).

#### 5-Lipoxygenase (5-LO)

Arachidonic acid (AA) is located in the phospholipid bilayer of the cell membrane and the precursor of main signal molecules. The metabolism of AA is closely associated with the development of cancer cells (78, 79). As a member of the arachidonic acid lipoxygenase family, 5-LO is composed of 674 amino acids and a monomeric enzyme containing iron ions. 5-LO can be transcriptionally regulated by t Egr, Sp1, nuclear factor-κB (NF-κB), and GATA (80).

5-LO is activated by 5-LO activating protein (ALOXAP) to catalyze AA which is released from the phospholipid bilayer by phospholipase A2. AA is transformed to 5-hydroxyeicosatetraenoic acid which can be metabolized by glutathione peroxidase into 5-hydroxyeicosatetranoic acid (5-HETE), which is further converted into either 5-oxo-eicosatetraenoic acid or LTA4. LTA4 is further converted into LTB4, LTC4, LTD4, or LTE4 depending on the different catalytic enzymes (81) (**Figure 1**). By immunohistochemistry, researchers found that the expression of 5-LO was high in epithelial ovarian cancer tissues and was associated with poor prognosis (35).

Z Wen et al. found that the high expression of 5-LO was strongly correlated with the density of TAMs in hypoxic areas of human ovarian tumor tissues. Leukotrienes (LTs) from 5-LO metabolites promoted migration and invasion of macrophages, which was mediated by up-regulation of matrix metalloproteinase-7 (MMP7) expression (35). Zileuton, a selective and specific 5-LO inhibitor, can reduce the expression of MMP-7 and the number of infiltrating macrophages in xenograft tumor tissues.

### Cyclooxygenase-2 (COX-2)

As another rate-limiting enzyme in AA metabolism, cyclooxygenase mainly catalyzes AA to produce prostaglandins (PGs) (**Figure 1**). Cyclooxygenase includes two isozymes, COX-1 and COX-2. COX1 maintains the homeostasis, while COX-2 can be induced by various stimulants, including cytokines, mitogens, hormones, and hypoxia. Growing evidence proves that COX-2 is highly expressed in cancers such as skin cancer, liver cancer, and breast cancer. Some studies reported that COX-2 and its derivative prostaglandin E2 (PGE2) were highly expressed in ovarian cancer cells and might promote cancer cell proliferation and metastasis (36, 82).

Angiogenesis is the physiological basis of solid cancer growth and metastasis. The high expression of COX-2 and its metabolite PGE2 promote angiogenesis through up-regulating of the angiogenic factors such as vascular endothelial growth factor (VEGF) and basic fibroblast growth factor (bFGF). COX-2 can also promote the metastasis and invasion of ovarian cancer through induction of matrix metalloproteinases (MMPs) in extracellular matrix and the decomposition of collagen matrix which may be involved in activation of the PI3K/AKT signaling pathway (83). Inhibition of COX-2 with its specific inhibitor NS-398 can increase the expression of E-cadherin and inhibit the expression of slug, vimentin, MMP2, and MMP9, thereby to suppress invasion and metastasis of ovarian cancer cells under estrogen treatment (84). Moreover, overexpression of COX-2 in ovarian cancer cells can directly up-regulate Bcl-2 expression through the increased synthesis of PGs. Celecoxib, a selective COX-2 inhibitor, can decrease cell growth, increase the cleaved caspase-3 activity and induce cell cycle G1 phase arrest in a dose-dependent manner in ovarian cancer cells (37).

## Exogenous Lipid Metabolism

The interactions between ovarian cancer cells and human peritoneal adipocytes in ascites are believed to be important for tumor progression. Co-culture of human primary omental adipocytes with ovarian cancer cells could transfer lipids directly from adipocytes to ovarian cancer cells, indicating that adipocytes may serve as an energy source for cancer cells (57).

### Fatty Acid Binding Protein 4 (FABP4)

The family of FABPs is a type of intracellular lipid chaperones that coordinate cellular lipid responses through binding to and redistributing intracellular fatty acids, so FABPs are also called lipid chaperone proteins (85). The function of FABP4 is to promote the uptake of long-chain fatty acids and to participate in lipid transport and metabolic regulation. Overexpression of FABP4 is reported in various types of tumors such as ovarian cancer. As a key mediator in adipocytes and cancer progression, FABP4 can be a worthy predictor of residual disease in ovarian cancer. Recent studies have found that miR-409-3p can target the 3’UTR region of FABP4 and regulate the expression of FABP4 (86).

Nieman et cal. compared primary ovarian cancers with corresponding omental metastatic tissues by immunohistochemical staining, and found that FABP4 was increased in ovarian cancer cells at the adipocyte-cancer interface, but was not detected in ovarian cancer cells and benign tissues adjacent to ovarian cancers far from the adipocyte-cancer interface (87). Co-culture of adipocytes with ovarian cancer cells showed that the adipocytes significantly promoted the metastasis of the ovarian cancer cells, whereas treatment of the co-cultured cells with FABP4 inhibitor, lipid accumulation and adipocyte-mediated invasion of the cancer cells were greatly reduced. In the latest research, knockdown of FABP4 in ovarian cancer cells resulted in the increasing level of 5-hydroxymethylcytosine, the downregulated expression of genes was associated with metastasis and the number of clone formation. BMS309403, a small molecule inhibitor of FABP4, was used and the results showed that it not only significantly reduced tumor burden in a syngeneic orthotopic mouse model but also increased the sensitivity of cancer cells towards carboplatin (39).

Taken together, these studies suggest that targeting FABP4 in ovarian cancer may inhibit the ability to adapt lipid-rich cancer microenvironment and to reduce tumor aggressiveness.

### CD36

CD36 is a transmembrane glycoprotein, which is one of the most abundantly expressed members in the class B scavenger receptor family. CD36 not only uptakes of free fatty acids and cholesterol, and the transfer of intracellular signals, but also pertains to the cancer-associated antigen presentation, inflammation, and angiogenesis (88). Studies have found that CD36 is highly expressed in ovarian cancer tissues and also metastatic tissues, which shows that CD36 may participate in the metastasis and proliferation of ovarian cancer.

Ladanyi et al. found that co-culture of ovarian cancer cells with human primary adipocytes (HPAs) increased the expression of CD36 in ovarian cancer cells. However, the inhibition of CD36 caused a decrease in fatty acid intake of cancer cells and reduced the accumulation of cholesterol and lipid droplets and the intracellular reactive oxygen species (ROS) in cancer cells. Knockdown of CD36 can also diminish adipocyte-mediated invasion and migration of cancer cells. Intraperitoneal injection of CD36-deficient cells significantly reduced the number of metastatic nodules in the abdominal of xenograft mouse tumor model (89). Thus, CD36 inhibition can effectively reduce fat acid uptake from microenvironment in ovarian cancer cells to suppress adipocyte-mediated tumor progression.

### Low Density Lipoprotein Receptor (LDLR)

LDLR is a trans-membrane protein that mediates the uptake of cellular cholesterol (90). Reports about LDLR mainly focus on the mechanism of LDLR-mediated chemo-resistance in ovarian cancer cells.

LDLR expression was reported to be correlated with the poor prognosis in patients with epithelial ovarian cancer (EOCs) treated with platinum-based drugs according to the cDNA chip database. Knockdown of LDLR can increase the sensitivity of cells to platinum, whereas overexpression of LDLR can promote chemotherapy resistance. The LDLR/LPC/FAM83B/FGFRs axis is involved in the LDLR-mediated resistance to platinum based chemotherapy. Zheng et al. determined that both SREBP2 and LDLR expression levels were increased in ovarian cancer cisplatin-resistant cell lines. Bioinformatics analysis predicts that SREBP2 may mediate ovarian cancer resistance through binding to LDLR (91).

### Lysophosphatidic Acid Receptor (LPAR)

Lysophosphatidic acid (LPA) is a kind of growth factor-like lipid signal molecule, and is secreted from platelets, nerve cells, and endothelial cells by endocrine and paracrine. LPA exerts its biological function through binding to the heterotrimeric transmembrane G protein coupled receptor (including Gα q, Gα 12/13, Gα i/o, and Gα s) on cell surface. At least six members of the receptor family are identified, named as LPA1-6 (92). These LPA receptors can be divided into two subfamilies, of which LPA1-3 are the member of vascular endothelial gene (edg) family and LPA4-6 belong to the family of non-vascular endothelial factors. LPA1 is widely distributed in heart, brain and kidney; LPA2 is distributed in testis, pancreas, and prostate; LPA3 is distributed in testis and prostate. All LPAs bind to cell surface receptors and are quickly degraded into inactive monoacylglycerol (MAG) and phosphatidic acid by phospholipase (93).

Ovarian cancer cells can uptake the lysophosphatidic acid through membrane receptors to promote proliferation (94). Studies have found that compared with normal ovarian tissues, LPA2 and LPA3 receptors were highly expressed in ovarian cancer tissues, while LPA1 receptor expression was still low. Inhibition of LPA2 or LPA3 receptor expression led to decreased cancer cell migration and invasiveness. Treatment of cells with LPA1 and LPA3 receptor-specific antagonist VPC32183 reduced the uptake of LPA and caused apoptosis through inhibition of the phosphorylation of ERK1/2. LPA and its receptors can regulate the promoter activity of cyclin D1 through the downstream signaling pathway of LTA receptor, which thereby promoting cell proliferation (95).

LPA and its receptors are also involved in cancer metastasis-related signaling pathways. Xu et al. found that the thyroid receptor interference protein 6 (TRIP6) can affect the LPA-induced cancer cell migration through directly binding to LPA2 receptor. The specific manifestation is that overexpression of TRIP6 enhanced the LPA-induced cell migration, while in contrast, inhibition of TRIP6 expression suppressed the LPA-induced cell migration, suggesting that TRIP6 may mediate the LPA2-induced cancer cell migration (96). Park et al. found that LPA could activate the downstream Gα 12/13/RhoA signaling pathway through LPA 1/2 receptor to induce the phosphorylation of ERM proteins (Ezrin/Radixin/Moesin), which promotes the metastasis of ovarian cancer cell line OVC-3 (97).

The combination of paclitaxel and cisplatin is a first-line chemotherapeutic strategy for ovarian cancer treatment. The researchers pretreated ovarian cancer cells with LPA followed by paclitaxel and found that LPA reduced mitochondrial ROS production while the LPA receptor agent VPC32183 increased the content of mitochondrial ROS. Further ROS could cause mitochondrial membrane damage and cancer cell apoptosis (98).

## Conclusion

Lipid metabolism of ovarian cancer is a complex process, including lipid uptake, lipid synthesis or storage, and fatty acid degradation by oxidation. So far, the researchers mainly clarified that the enzymes related to fatty acid synthesis (FASN, ACC, ACLY, SCD) and lipid degradation related enzymes (MAGL, CPT, 5-LO, COX2), and receptors related to lipid uptake (FABP4, CD36, LDLR) play important roles in promoting cancer development (**Figure 1**). However, the study of lipid metabolomics for ovarian cancer markers is still in the primary stage.

In this review, we systematically summarized the process metabolism of fatty acid and the rate-limiting enzymes in this framework. Meanwhile, a number of promising agents targeting the lipid metabolism axis are being developed and applied in clinical treatment, which can provide new strategies for clinical treatment of ovarian cancer.

## Author Contributions

GY and XZ were responsible for the revision of the manuscripts. ZJ completed the writing. YShe and XF were involved in the design of the manuscripts. JM, YSha, and YK completed the documentation and figure drawing. All authors contributed to the article and approved the submitted version.

## Conflict of Interest

The authors declare that the research was conducted in the absence of any commercial or financial relationships that could be construed as a potential conflict of interest.

## Supplementary Material

Supplementary Table 1 Abbreviation List. AA, arachidonic acid; ACC, acetyl-CoA carboxylase; ACLY, ATP citrate lyase; CACT, carnitine-acylcarnitine translocase; CIC, citrate carrier protein; COX2, cyclooxygenase-2; CPT1, carnitine palmitoyl transferase 1; CPT2, carnitine palmitoyl transferase 2; DGAT, diacylglycerol acyltransferase; ELOVL, elongation of very long-chain fatty acids diacylglycerol acyltransferase; FADS, fatty acid desaturase; FSAN, fatty acid synthase; FABP4, fatty acid binding protein 4; LDLR, low density lipoprotein receptor; LTA4, lipoxin A4; LTB4, leukotriene B4; LTC4, leukotriene C4; LTD4, leukotriene D4; LTE4, leukotriene E4; LPAR, lysophosphatidic acid receptor; MAGL, monoacylglycerol lipase; MCD, malonyl-CoA decarboxylase; MG, monoglyceride; MUFA, monounsturated fatty acids; PGD2, prostaglandin D2; PGE2, prostaglandin E2; PGF2, prostaglandin F2; PGH2, prostaglandin H2; PGI2, prostaglandin I2; PLA2, phospholipase A2; PUFA, polyunsturated fatty acids; SCD1, stearoyl COA desaturase1; SFA, saturated fatty acids; TxA2, thromboxane A2; 5-HETE, 5-hydroxyeicosatetraenoic acid; 5-HPETE, 5-hydroperoxy-eicosatetraenoic acid; 5-LO, 5-Lipoxygenase.
