# Supplementary material for: Deregulation of Lipid Metabolism: The Critical Factors in Ovarian Cancer
Source: Front Oncol. 2020 Oct 19;10:593017. doi: 10.3389/fonc.2020.593017 (PMC7604390; doi:10.3389/fonc.2020.593017)
Supplement: Supplementary Table 1 — Abbreviation List. AA, arachidonic acid; ACC, acetyl-CoA carboxylase; ACLY, ATP citrate lyase; CACT, carnitine-acylcarnitine translocase; CIC, citrate carrier protein; COX2, cyclooxygenase-2; CPT1, carnitine palmitoyl transferase 1; CPT2, carnitine palmitoyl transferase 2; DGAT, diacylglycerol acyltransferase; ELOVL, elongation of very long-chain fatty acids diacylglycerol acyltransferase; FADS, fatty acid desaturase; FSAN, fatty acid synthase; FABP4, fatty acid binding protein 4; LDLR, low density lipoprotein receptor; LTA4, lipoxin A4; LTB4, leukotriene B4; LTC4, leukotriene C4; LTD4, leukotriene D4; LTE4, leukotriene E4; LPAR, lysophosphatidic acid receptor; MAGL, monoacylglycerol lipase; MCD, malonyl-CoA decarboxylase; MG, monoglyceride; MUFA, monounsturated fatty acids; PGD2, prostaglandin D2; PGE2, prostaglandin E2; PGF2, prostaglandin F2; PGH2, prostaglandin H2; PGI2, prostaglandin I2; PLA2, phospholipase A2; PUFA, polyunsturated fatty acids; SCD1, stearoyl COA desaturase1; SFA, saturated fatty acids; TxA2, thromboxane A2; 5-HETE, 5-hydroxyeicosatetraenoic acid; 5-HPETE, 5-hydroperoxy-eicosatetraenoic acid; 5-LO, 5-Lipoxygenase. [file Table_1.docx]

**Table S1 Abbreviation List**

AA: arachidonic acid

ACC: acetyl-CoA carboxylase

ACLY: ATP citrate lyase

CACT: carnitine-acylcarnitine translocase

CIC: citrate carrier protein

COX2: cyclooxygenase-2

CPT1: carnitine palmitoyl transferase 1

CPT2: carnitine palmitoyl transferase 2

DGAT: diacylglycerol acyltransferase

ELOVL: elongation of very long-chain fatty acids diacylglycerol acyltransferase

FADS: fatty acid desaturase

FSAN: fatty acid synthase

FABP4: fatty acid binding protein 4

LDLR: low density lipoprotein receptor

LTA4: lipoxin A4

LTB4: leukotriene B4

LTC4: leukotriene C4

LTD4: leukotriene D4

LTE4: leukotriene E4

LPAR: lysophosphatidic acid receptor

MAGL: monoacylglycerol lipase

MCD: malonyl-CoA decarboxylase

MG: monoglyceride

MUFA: monounsturated fatty acids

PGD2: prostaglandin D2

PGE2: prostaglandin E2

PGF2: prostaglandin F2

PGH2: prostaglandin H2

PGI2: prostaglandin I2

PLA2: phospholipase A2

PUFA: polyunsturated fatty acids

SCD1: stearoyl COA desaturase1

SFA: saturated fatty acids

TxA2: thromboxane A2

5-HETE: 5-hydroxyeicosatetraenoic acid

5-HPETE: 5-hydroperoxy-eicosatetraenoic acid

5-LO: 5-Lipoxygenase
